# Identification of pneumococcal colonization determinants in the stringent response pathway facilitated by genomic diversity

**DOI:** 10.1186/s12864-015-1573-6

**Published:** 2015-05-09

**Authors:** Yuan Li, Nicholas J Croucher, Claudette M Thompson, Krzysztof Trzciński, William P Hanage, Marc Lipsitch

**Affiliations:** Department of Epidemiology, Center for Communicable Disease Dynamics, Harvard T.H. Chan School of Public Health, Boston, Massachusetts USA; Department of Immunology & Infectious Diseases, Harvard T.H. Chan School of Public Health, Boston, Massachusetts, 02115 USA; Department of Pediatric Immunology and Infectious Diseases, UMC Utrecht, WKZ, Lundlaan 6, 3508 AB Utrecht, The Netherlands

**Keywords:** Genomic diversity, Pneumococcal colonization, RelA/ SpoT homolog

## Abstract

**Background:**

Understanding genetic determinants of a microbial phenotype generally involves creating and comparing isogenic strains differing at the locus of interest, but the naturally existing genomic and phenotypic diversity of microbial populations has rarely been exploited. Here we report use of a diverse collection of 616 carriage isolates of *Streptococcus pneumoniae* and their genome sequences to help identify a novel determinant of pneumococcal colonization.

**Results:**

A spontaneously arising laboratory variant (SpnYL101) of a capsule-switched TIGR4 strain (TIGR4:19F) showed reduced ability to establish mouse nasal colonization and lower resistance to non-opsonic neutrophil-mediated killing *in vitro*, a phenotype correlated with *in vivo* success. Whole genome sequencing revealed 5 single nucleotide polymorphisms (SNPs) affecting 4 genes in SpnYL101 relative to its ancestor. To evaluate the effect of variation in each gene, we performed an *in silico* screen of 616 previously published genome sequences to identify pairs of closely-related, serotype-matched isolates that differ at the gene of interest, and compared their resistance to neutrophil-killing. This method allowed rapid examination of multiple candidate genes and found phenotypic differences apparently associated with variation in SP_1645, a RelA/ SpoT homolog (RSH) involved in the stringent response. To establish causality, the alleles corresponding to SP_1645 were switched between the TIGR4:19F and SpnYL101. The wild-type SP_1645 conferred higher resistance to neutrophil-killing and competitiveness in mouse colonization. Using a similar strategy, variation in another RSH gene (TIGR4 locus tag SP_1097) was found to alter resistance to neutrophil-killing.

**Conclusions:**

These results indicate that analysis of naturally existing genomic diversity complements traditional genetics approaches to accelerate genotype-phenotype analysis.

**Electronic supplementary material:**

The online version of this article (doi:10.1186/s12864-015-1573-6) contains supplementary material, which is available to authorized users.

## Background

A general question in microbiology is to understand the genetic determinants underlying a microbial phenotype, such as increased virulence or transmissibility. Developments in sequencing technology now allow rapid identification of genetic differences among phenotypic variants, but it remains a time-consuming process to verify the function of each genetic variation by constructing isogenic strains (chimeras) in which only one gene of interest differs. Genomic diversity of existing microbial populations can be used to infer the consequences of polymorphisms by evaluating phenotypic differences between naturally occurring variants, if 1) the naturally occurring variants harbor the genetic variation of interest and 2) other known genetic determinants of the phenotypic difference can be accounted for. Testing existing variants can reduce the amount of time and resources spent on constructing isogenic strains, so we hypothesized that using naturally occurring genomic diversity can accelerate gene function discovery.

*Streptococcus pneumoniae* (pneumococcus) is an important human pathogen that causes pneumonia, meningitis, sinusitis and otitis media worldwide. Pneumococcus frequently colonizes the human nasopharynx, which precedes invasive infections, and these colonization events serve as the reservoir for bacterial transmission [1]. There are more than 90 capsular serotypes of pneumococcus. Simultaneous colonization by multiple serotypes and competition between serotypes has been documented during nasopharyngeal carriage in humans [1,2]. Understanding the factors that determine serotype patterns of carriage is an important public health issue [3,4]. The capsule itself has a major impact on carriage: studies have shown that serotype controls resistance to surface killing mediated by human neutrophils [4]*,* ability to compete against co-colonizing strains in a mouse model of multiple-strain carriage [Krzysztof Trzciński et al., unpublished data], and pneumococcal cell surface charge [5]. All the three properties, in turn, are correlated with carriage prevalence in human populations [4,5]. For genetic variations outside the capsular polysaccharide synthesis (*cps*) region, much less is known about their contribution to resistance to surface killing or ability to compete during nasopharyngeal colonization.

**Figure 1:**
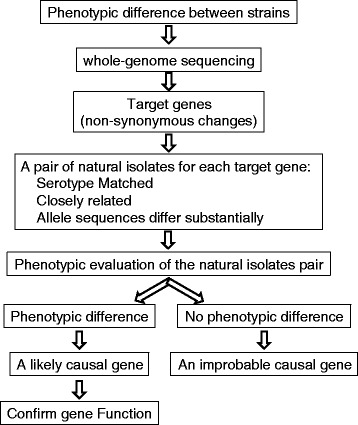
A diagram summarizing the approach to use naturally existing variants in combination with sequencing of laboratory variants to accelerate the discovery of genetic variation underlying phenotypes of interest

Here we report an approach (Figure 1) that uses existing genomic diversity within a collection of carriage isolates, for which draft genomes are available, to accelerate the discovery of a novel, non-capsule genetic determinant of pneumococcal colonization. The project began during an experimental study of competitive colonization of mice by different strains of pneumococci, which had been constructed such that they were all of the TIGR4 genetic background but differed at the *cps* locus, which determines serotype. A spontaneous variant of one of these strains, TIGR4:19F (hereafter the variant is called SpnYL101), showed an unexpected but repeatable change of phenotype, specifically a reduced ability to colonize a mouse in the presence of competing strains, relative to the parental strain. We sequenced the whole genomes of the parental strain and the variant and identified 5 single nucleotide polymorphisms (SNPs) separating them. To improve the efficiency of the effort to identify which of these SNPs had caused the phenotypic change, we set out to evaluate the effect of sequence variation in genes that are affected by the SNPs by testing a related phenotype in pairs of isolates selected from a genomically sequenced collection of pneumococci carried by children in Massachusetts. For each candidate gene, we sought pairs of isolates from the sequenced collection that (1) were serotype-matched, because serotype is a known determinant of competitive colonization; (2) diverged substantially at the candidate gene, and (3) were otherwise as similar genetically as possible. We hypothesized that within an isolate pair selected by these criteria, phenotypic differences could be reasonably attributed to sequence variations in the gene that differed substantially. After examining three such pairs, we found evidence supporting the role of variation in gene SP_1645, a RelA/SpoT homolog (RSH) protein involved in the stringent response (SR). The SR globally regulates bacterial transcription, translation and replication in response to nutrient limitation and other stresses [6]. The RSH proteins in Gram-positive bacteria catalyze the synthesis and degradation of guanosine tetraphosphate (ppGpp), which is a critical mediator of the stringent response [7]. The RelA homolog protein in pneumococcus (encoded by *rel*_spn_) was previously shown to modulate pneumococcal disease severity and course of progression in a mouse model of infection [8]. We confirmed the effect of the SP_1645:T1019C variation, one of the 5 SNPs separating TIGR4:19F and SpnYL101, by evaluating the phenotypes in strains in which the SP_1645 alleles were exchanged. Using a similar strategy, we found that variation in another RSH gene (SP_1097) contributes to change in resistance to neutrophil-killing. As collections of bacterial whole genome sequence data become larger, they will provide an increasingly valuable resource for targeting candidate genetic changes that cause phenotypic changes of interest.

## Results

### A variant of pneumococcus with reduced fitness

**Figure 2:**
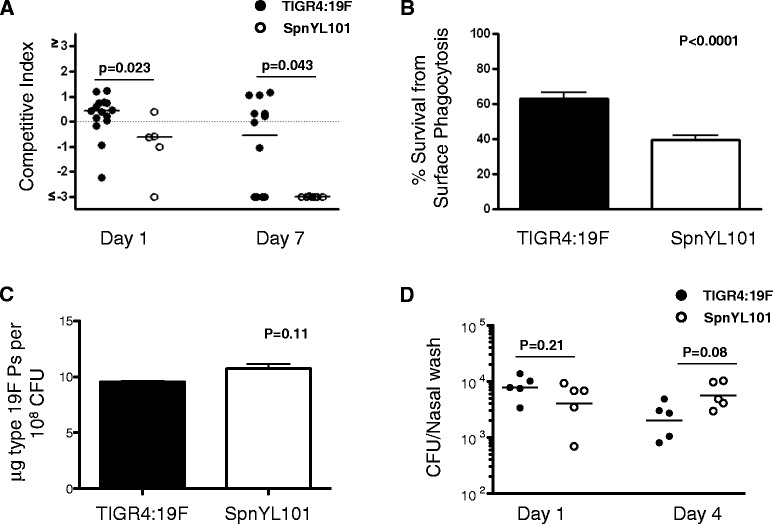
A variant of the TIGR4:19F strain, SpnYL101, showed impaired colonization in a mouse model of multi-strain nasopharyngeal carriage and lower resistance to surface phagocytosis *in vitro.* **(A)** In a mouse colonization model, five TIGR4 capsular variants (TIGR4:1, TIGR4:4, TIGR4:14, TIGR4:19A, TIGR4:23F) were mixed with either TIGR4:19F (solid circle) or SpnYL101 (open circle) at equal amount (~10^6^ CFU each) and were used to intranasally challenge C57BL/6 mice. Nasal wash samples were obtained on days 1 and 7 post challenge, and the frequency of the serotype 19 F variant in each sample was determined by qPCR. The competitive index of the TIGR4:19F or SpnYL101 in each sample is shown. Horizontal bars are medians. p-values are derived from Mann–Whitney tests. **(B)** Survival rate of the TIGR4:19F and the SpnYL101 measured in a non-opsonic neutrophil-mediated killing assay. Error bars represent SEM. The p-value is derived from t-test (n = 10 for each group). **(C)** Cell-associated type 19 F capsular polysaccharide (Ps) measured by an inhibition ELISA assay. Error bars represent SEM from two independent experiments. The p-value is derived from t-test. **(D)** Single-strain colonization of C57BL/6 mice by either TIGR4:19F or SpnYL101. Colonization densities as measured by total CFU/nasal wash on day 1 and day 4 are shown. Horizontal bars are medians. p-values are derived from Mann–Whitney tests.

We observed a variant of the TIGR4:19F strain (SpnYL101) showing reduced ability to compete against other strains during nasal colonization, compared to the originally constructed TIGR4:19F (Figure 2A). When co-colonizing C57BL/6 mice together with 5 other strains (TIGR4:1, TIGR4:4, TIGR4:14, TIGR4:19A, and TIGR4:23F), the SpnYL101 strain showed significantly lower competitive index on day 1 (median = −0.63) than did the originally constructed TIGR4:19F strain (median = 0.42; Mann–Whitney test, U = 11.00, n_1_ = 15, n_2_ = 5, p = 0.023). By day 7, the competitive index of the SpnYL101 strain was close to the lower limit of detection (median = −3.00), which was significantly lower than the competitive index of the TIGR4:19F strain (median = −0.55; Mann–Whitney test, U = 24.00, n_1_ = 14, n_2_ = 7, p = 0.043). The results indicate that the SpnYL101 strain is less likely to establish carriage in mice than the TIGR4:19F strain in the presence of co-colonizers.

It has been reported that higher carriage prevalence of *S. pneumoniae* in human populations is associated with higher resistance to neutrophil-killing [4]. We therefore tested whether the SpnYL101 strain is more likely to be killed by neutrophils than the TIGR4:19F strain in a surface killing assay [4]. As shown in Figure 2B, the survival of the SpnYL101 strain (mean = 0.39, SD = 0.084) was significantly lower than that of the TIGR4:19F strain (mean = 0.60, SD = 0.11; t-test, t(16) = −5.28, p < 0.0001).

Decreased degree of encapsulation was shown to reduce *S. pneumoniae* resistance to non-opsonic neutrophil-mediated killing [4]. We therefore assessed whether the SpnYL101 strain exhibited reduced capsule production compared to TIGR4:19F. Both strains were confirmed to remain serotype 19 F by Pneumotest-latex serotyping. The cell-associated type 19 F capsular polysaccharide was quantified by an inhibition ELISA assay, and no evidence of reduction in capsule production was observed for SpnYL101 (Figure 2C). Furthermore, SpnYL101 and TIGR4:19F showed similar efficiency in colonizing C57BL/6 mice in single-strain colonization experiment (Figure 2D). Thus, the SpnYL101 strain appeared to have acquired non-capsule change(s) that can affect both competition during co-colonization and resistance to surface phagocytosis. We measured the *in vitro* growth rates of SpnYL101 and TIGR4:19F in THY, and no statistically significant difference was observed (Additional file 1: Figure S1).

### Genetic Variations identified in the SpnYL101 strain

We hypothesized that the phenotypic differences between the SpnYL101 and the TIGR4:19F strains were caused by genetic variation. To test this hypothesis, we sequenced the whole genome of the two strains using the Illumina MiSeq system and compared the two genomes to identify SNPs distinguishing the two strains.

Five SNP sites were identified between the SpnYL101 and the TIGR4:19F genomes (Table 1). Four SNP sites were located in coding sequences (CDS) and the other one was located in an intergenic region. Four genes, with TIGR4 locus tags SP_0152, SP_0655, SP_1119, and SP_1645, were affected by the four SNPs located in CDS, respectively. Consistent with our serotyping result, no SNPs were located in the *cps* locus that is responsible for capsular polysaccharide synthesis. In the SP_0655 locus, the SNP did not alter the amino acid sequence encoded (synonymous SNP), and therefore it was not likely to cause a phenotypic difference. In contrast, in the SP_0152, SP_1119, and SP_1645 loci, the SNP caused a change in the translated amino acid sequence (non-synonymous SNP). These three single-nucleotide variations might explain the differing phenotypes of the TIGR4:19F and SpnYL101 strains.

**Table 1:** **SNPs identified between TIGR4:19F and SpnYL101**

**Position** ^**a**^	**TIGR4:19F**	**SpnYL101**	**CDS**	**Codon** ^**b**^	**SNP Name** ^**c**^
150259	A	C	SP_0152	N	SP_0152:C380A
626569	C	T	SP_0655	S	SP_0655:C906T
1052463	A	G	SP_1119	N	SP_1119:G652A
1542955	G	A	SP_1645	N	SP_1645:C1019T
1543998	G	A	-	I	A1543998G

^a^Position corresponds to reference TIGR4 genome sequence (GenBank accession: AE005672).

^b^Indicator of the effect of the single nucleotide difference: non-synonymous codon change (N), synonymous codon change (S), or change in the inter-gene region (I).

^c^SNP located in a CDS is named according to the CDS name. After the CDS name, the first letter is the nucleotide sequence in the coding strand of the TIGR4 genome; the number represents the nucleotide location within the CDS; and the second letter represents the alternative nucleotide sequence. SNP located in the inter-gene region is named by a letter (nucleotide sequence in the TIGR4 genome), followed by a number (the SNP position) and another letter (the alternative nucleotide sequence).

### Testing the effects of SNP with naturally existing diversity

To test whether variation in a candidate gene causes a phenotypic difference, classical genetic approaches involve creating a set of isogenic strains in which only the candidate gene differs and evaluating phenotypes among the set of isogenic strains. If multiple candidate genes were to be examined, multiple sets of isogenic strains would usually need to be constructed.

**Figure 3:**
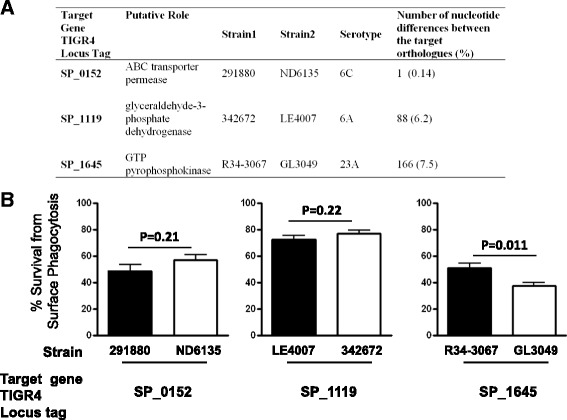
Evidence for the effects of variable genes between TIGR4:19F and SpnYL101 in carriage isolates. **(A)** For each gene that contained a non-synonymous substitution between TIGR4:19F and SpnYL101, we identified a pair of carriage isolates that are serotype-matched, closely related, but differ substantially in the target gene alleles, such that phenotypic differences within the pair could be reasonably attributed to allelic variations in the target gene. **(B)** The carriage isolates were subjected to the non-opsonic neutrophil-mediated killing assay. Mean survival rates are shown (n = 10 for each strain). Error bars represent SEM. The p-values were derived from t-tests.

We sought to accelerate this process using a diverse collection of nasopharyngeal carriage isolates for which draft whole-genome sequences have previously been obtained [9]. We first examined whether genes affected by the non-synonymous SNPs between the SpnYL101 and the TIGR4:19F also vary in the carriage isolates. Sequence variation was found for all the three genes affected by non-synonymous SNPs (Figure 3A). For each target gene, we identified a pair of serotype-matched, closely-related isolates that differed substantially at the target gene (Figure 3A, see Materials and Method for identification protocol). Thus, each pair of carriage isolates mimicked a set of quasi-“isogenic” strains and was subsequently tested in the surface killing assay. Comparatively little variation was found around the rest of the genome (Additional file 1: Table S1). As shown in Figure 3B, no significant difference in resistance to neutrophil-killing was found between isolates 291880 and ND6135 (designed to test effects of sequence variation in SP_0152; t-test, t(18) = 1.30, p =0.21) or between isolates 342672 and LE4007 (designed to test effects of sequence variation in SP_1119; t-test, t(18) = 1.29, p =0.22). In contrast, a significant difference in resistance to neutrophil-killing was found between isolates R34-3067 and GL3049 (designed to test effects of sequence variation in SP_1645; t-test, t(18) = 2.82, p =0.011). A whole genome alignment of this pair found only 28 sites at which these two isolates had different bases [9]. These results suggested that the SP_1645 variation might be a non-capsule determinant of resistance to neutrophil-killing.

### The SP_1645 C1019T variation in SpnYL101 contributes to its defects

**Figure 4:**
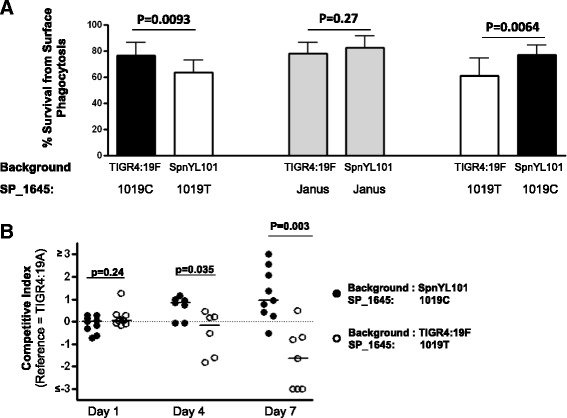
The SP_1645:C1019T variation leads to lower resistance to surface phagocytosis in vitro and impaired competitiveness *in vivo*. **(A)** The TIGR4:19F strain exhibited higher resistance to neutrophil-mediated killing than the SpnYL101 strain (left two bars). The SP_1645 gene was replaced by a Janus cassette in the TIGR4:19F and the SpnYL101, and the resulting SP_1645- knock out strains showed no significant difference in resistance to neutrophil-killing (middle two bars). The TIGR4:19F strain carrying SP_1645 1019 T allele (SpnYL104) was less resistant to neutrophil- killing than did the SpnYL101 strain carrying the SP_1645 1019C allele (SpnYL105, right two bars). Error bars represent SEM. The p-values were derived from t-tests (n = 10 for each group). **(B)** Competitive index of the SpnYL104 (open circles) and the SpnYL105 (solid circles) relative to a capsular variant of the TIGR4 (TIGR4:19A) as the reference strain. Data on days 1, 4 and 7 post-challenge are shown. The horizontal bars are medians. p-values are derived from Mann–Whitney tests.

To confirm whether the SP_1645:C1019T variation in SpnYL101 decreases the resistance to neutrophil-killing *in vitro*, we first replaced the SP_1645 locus in both the TIGR4:19F and the SpnYL101 by a Janus cassette to knock out the gene function. Unlike the parental strains, the SP_1645-knock out strains showed no significant difference in survival from neutrophil-killing (Figure 4A, middle two bars, t(17) = 1.15, p = 0.27). The results suggested that the SP_1645 gene is necessary for this phenotypic difference between TIGR4:19F and SpnYL101. Subsequently, we switched the alleles the SP_1645 locus between the TIGR4:19F and the SpnYL101 strains by transforming the SP_1645-knock out strains of TIGR4:19F and SpnYL101 with alleles amplified from the SP_1645 locus of SpnYL101 and TIGR4:19F, respectively. The resulting two strains, SpnYL104 and SpnYL105, were subjected to the surface killing assay (Figure 4A, right two bars). The survival of SpnYL104 (mean = 0.61 S.D. =0.14) was significantly lower than that of SpnYL105 (mean = 0.76, S.D. = 0.08; t(14) = 3.21, p = 0.0064). The results indicated that the allele in the SP_1645 locus of the SpnYL101 strain is sufficient to confer reduced resistance to neutrophil-killing *in vitro*.

Since the SP_1645 SNP caused a change in resistance to neutrophil-killing, we hypothesized that the SP_1645:C1019T variation also contributes to the ability to establish nasal colonization. To test this hypothesis, a reference strain (TIGR4:19A) was mixed with either SpnYL104 or SpnYL105 at equal ratio and the mixtures were used to intranasally challenge C57BL/6 mice. Nasal wash samples were collected on days 1, 4, and 7 post-challenge, and the competitive index was measured as described in Materials and Methods. The SpnYL104 and the SpnYL105 showed similar competitive index on day 1 (Figure 4B, Mann–Whitney test, U = 30.00, n_1_ = 9, n_2_ = 10, p = 0.24). By day 4, the SpnYL105 strain showed a slightly higher competitive index (median = 0.86) than was observed for the SpnYL104 (median = −0.17) and the difference was significant (Mann–Whitney test, U = 6.00, n_1_ = 7, n_2_ = 6, p = 0.035). By day 7 of colonization, the competitive index for the SpnYL105 strain (median = 0.94) was significantly higher than that of the SpnYL104 strain (median = −1.63, Mann–Whitney test, U = 3, n_1_ = 9, n_2_ = 7, p = 0.003). The results indicated that the SP_1645:C1019T variation is an important determinant of pneumococcal competitiveness during nasal colonization in the mouse.

### Effects of variation in other genes involved in the stringent response pathway

**Figure 5:**
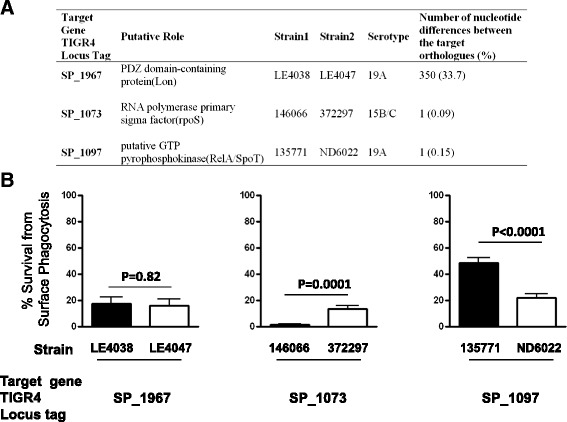
Evidence for variation of specific stringent response genes associated with change in resistance to neutrophil-killing. **(A)** Pneumococcal genes assumed to be involved in the stringent response pathway were identified by searching the KEGG Orthology groups that contain stringent response genes in E.coli (K07177, K03086, and K07816). To test the effects of sequence variation in a target gene, a pair of pneumococcal carriage isolates that are serotype-matched, closely related, but differ substantially at the target gene were identified. **(B)** The carriage isolates were subjected to the non-opsonic neutrophil-mediated killing assay. Mean survival rates are shown (n = 10 for each strain). Error bars represent SEM. The p-values were derived from t-tests.

The SP_1645 gene product belongs to the RelA/SpoT homolog proteins mediating the stringent response (SR). It is of interest to understand the role of the pneumococcal SR pathway in colonization success. We hypothesized that naturally existing genomic diversity can provide information on the effects of variation in other SR genes. Three putative pneumococcal SR genes (TIGR4 locus tag SP_1967, SP_1073, and SP_1097) were identified according to the KEGG Orthology database (K07177, K03086, and K07816, respectively). For each putative SR gene, we identified a pair of serotype-matched, closely-related isolates that differ substantially at the target gene (Figure 5A) and evaluated their survival from surface phagocytosis (Figure 5B). No significant difference in survival from surface phagocytosis was found between isolates LE4038 and LE4047 (designed to test effects of sequence variation in the gene with TIGR4 locus tag SP_01967; t-test, t(18) = 0.23, p = 0.82). In contrast, a significant difference in resistance to neutrophil-killing was found between isolates 146066 and 372297 (designed to test effects of sequence variation in SP_1073; t-test, t(18) = 4.90, p =0.0001) and between isolates 135771 and ND6022 (designed to test effects of sequence variation in SP_1097; t-test, t(18) = 5.21, p < 0.0001). The results suggested that variation in specific stringent response genes was associated with change in resistance to neutrophil-killing.

**Figure 6:**
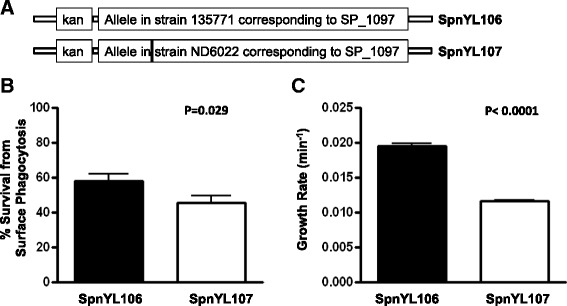
Mutation in SP_1097 results in higher resistance to surface phagocytosis and higher growth rate *in vitro*. **(A)** The SP_1097 locus in the TIGR4:6B strain was replaced by a kanamycin resistance marker (Kan) fused to the allele corresponding to SP_1097 from either strain 135771 or strain ND6022 to construct the SpnYL106 and SpnYL107 strains, respectively. The vertical black bar indicates the only SNP between the two alleles (C for 135771 and T for ND6022 at position 170). **(B)** Survival rate of the SpnYL106 and the SpnYL107 strains measured in a non-opsonic neutrophil-mediated killing assay. Error bars represent SEM. The p-value is derived from t-test (n = 10 for each group). **(C)** Growth rate of the SpnYL106 and the SpnYL107 strains. The strains were cultured in THY medium at 37°C and the increase in O.D. 620 was measured for 6 hours. The increase in O.D. was fitted to an exponential growth to estimate the growth rate. Error bars represent SEM. The p-value is derived from t-test (n = 12 for each group).

SP_1097 is the only other gene in the TIGR4 genome that encodes a putative GTP pyrophosphokinase [EC:2.7.6.5] in addition to SP_1645. GTP pyrophosphokinase activity could influence the level of guanosine tetraphosphate (ppGpp), which is the upstream mediator of SR and controls other downstream SR effectors. We therefore wanted to test whether the difference between alleles corresponding to SP_1097 in 135771 and ND6022 (only one non-synonymous SNP) causes changes in resistance to neutrophil-killing. We constructed two isogenic strains, SpnYL106 and SpnYL107 (Figure 6A), in which the SP_1097 locus in the TIGR4:6B was replaced by alleles corresponding to SP_1097 in 135771 and ND6022, respectively. Consistent with what was observed for carriage isolates 135771 and ND6022, the SpnYL106 showed a significantly higher survival than the SpnYL107 (Figure 6B; t-test, t(18) = 2.38, p =0.029). We also estimated the *in vitro* growth rate for both strains by fitting growth curves to an exponential growth equation. The SpnYL106 showed a significantly higher *in vitro* growth rate than the SpnYL107 (Figure 6C; t-test, t(14) = 20.3, p <0.0001). To test whether the SP_1097 SNP influences colonization *in vivo*, the competitive indexes of SpnYL106 and SpnYL107 were measured as described in Materials and Methods. Among mice that remained detectably colonized by at least one strain, SpnYL106 and SpnYL107 showed similar competitive indexes on both day 1 and day 4 (Additional file 1: Figure S2).

## Discussion

In this study, we observed phenotypic differences between the TIGR4:19F and SpnYL101 strains. We sequenced the whole genomes of the two strains and identified 5 SNPs as the candidate causes of the phenotypic differences. Subsequently, we prioritized hypotheses about the likely roles of genetic variation in genes affected by a SNP by testing a pair of serotype-matched, closely-related carriage isolates that differs substantially at each candidate gene. Such isolate pairs were identified by genomic analysis of a large collection of carriage isolates with diverse genetic background and evidence was found to support the phenotypic impact of variation in the gene with TIGR4 locus tag SP_1645. Although it would be ideal to compare closely-related isolate pairs that harbor the exact same SNP of interest, such pairs may not always be available for a given collection (Additional file 1: Table S2). Additionally, this method aims to make the best use of the available genomic diversity information to complement (not replace) traditional genetics approaches and accelerate genotype-phenotype analysis. It could be inappropriate if the distance between the most closely related strains happens to be quite large because more genomic changes would make it difficult to evaluate the contribution from a specific gene variation. We also acknowlodge that serotype matching may not be as informative for other studies. As each pair of isolates came from the same ‘sequence cluster’, there is unlikely to be much relevant accessory genome variation distinguishing them [10], making it unlikely that the differences in phenotypes are down to other genetic variation, althogh such differences cannot be formally ruled out. We therefore emphasize that the method is complementary to, rather than a direct replacement for, experimental work that can identify the causal genetic variant underlying phenotypic differences. Finally, we confirmed the effects of the SP_1645:C1019T variation by using laboratory-constructed isogenic strains. Notably, the TIGR4:19F and SpnYL101 strains became similarly susceptible to neutrophil-killing when the SP_1645 locus was knocked out from both strains (Figure 4A). This result indicated that the other 4 SNPs were unlikely to play a major role in resistance to neutrophil-killing.

Little is known about the cellular function of the SP_1645:C1019T variation we identified. When the SP_1645 locus in the TIGR4:19F strain was replaced by the Janus cassette, no significant change in resistance to neutrophil-killing was observed. This is consistent with results in a previous transposon mutagenesis study [11], also using variants of TIGR4 (presumably with the wild-type allele), that disruption of the SP_1645 locus by transposon insertions did not cause a detectable defect in pneumococcal colonization of mouse nasopharynx. In contrast, replacing the SP_1645 locus in the SpnYL101 strain by Janus cassette significantly increased resistance to neutrophil-killing, restoring it to a level similar to that seen in the TIGR4:19F strain. This observation suggested that the SP_1645 allele in SpnYL101 could represent a gain-of-function change in which the gained function appeared to associate with reduced resistance to surface killing. Since it has been shown that surface charge of pneumococcus influences its resistance to neutrophil-killing [5], we measured the zeta potential of the TIGR4:19F, SpnYL101, and the SP_1645 knock-out strains, and did not find a significant difference in surface charge among them. In addition, the protein encoded by the SP_1645 gene was not in a previously reported list of human antibody antigens [12] or a list of human T_H_-17 cell antigens [13]. Thus, the SP_1645:C1019T variation could represent a previously unknown determinant that, directly or indirectly, influences the interaction between pneumococcus and neutrophils.

The gene with TIGR4 locus tag SP_1645 is inferred to encode a RelA/SpoT homolog protein, which is involved in the synthesis and degradation of guanosine tetraphosphate (ppGpp). In bacteria, ppGpp is a stringent-response mediator that coordinates cellular activities in response to changes in nutrient abundance [14,15]. We therefore tested the effects of variation in other genes involved in the stringent response pathway using, once again, naturally existing genomic diversity. Interestingly, evidence suggested that variation in another RelA/SpoT homolog protein encoded by the gene with TIGR4 locus tag is also associated with change in resistance to neutrophil-killing. Construction and testing isogenic strains further confirmed the causal effects of the SNP in the gene with TIGR4 locus tag SP_1097. These data suggested that RelA/SpoT homolog proteins in pneumococcus could play a critical role in regulating bacterial responses to neutrophil-killing. Interestingly, no significant difference in competitiveness against SpnYL049 observed between SpnYL106 and SpnYL107 in the mouse colonization experiment (Additional file 1: Figure S2). The results indicated that, consistent with previous studies [4,5], resistance to phagocytic killing is an important but not the only mechanism to regulate pneumococcal colonization ability. It should be noted that mouse colonization *in vivo* and surface phagocytosis killing *in vitro* are two complementary, relevant, if imperfect model systems we used to understand the pneumococcal determinants of colonization success in human populations. More studies using a variety of approaches are needed to dissect the possible links between activities of GTP pyrophosphokinase (including gene products of SP_1645 and SP_1097), levels of stringent response, and pneumococcal colonization success.

The increasing availability of large, publicly available genomic datasets, combined with relatively inexpensive sequencing of laboratory variants, provides a wealth of information that can be mined for polymorphisms with interesting phenotypic consequences. In addition to whole genome sequences, databases on transcriptomics, proteomics, protein structure, and specific functions (e.g. antibiotic resistance, immunogenicity) could also be mined to obtain information on potential role of the targets, and therefore help focusing on genetic variations of high likelihood of functional consequences. The approach used here requires access to enable us to search such data for suitably matched, closely related isolates distinguished by the polymorphism of interest, and then to the selected isolates themselves for phenotypic evaluation. In this study we used carriage isolates to which we already had access and their whole genome sequences, which are not trivial to obtain. In order to facilitate more general use at the public level, affordable publicly funded repositories (e.g. the BEI resources http://www.beiresources.org/) should be supported. The option to use an *in silico* search of genomic sequence database to identify comparable isolates in which the SNP has already occurred in the course natural evolution can be far more efficient than constructing an isogenic mutant *in vitro*. This method allows us to quickly screen multiple SNPs and narrow down a candidate list to those with high likelihood of causing a phenotypic difference. The same procedure can be applied to studying the effect of genetic variation in other microorganisms for which collections of diverse isolates and their full-genome sequences are available. It will be particularly useful when gene replacement and creation of isogenic strains are difficult to achieve, such as in studies of essential genes or microorganisms with low recombination rate. Given the fast growing database of whole genome sequencing data for many different organisms [16,17], a method that uses naturally existing diversity of microbial populations to accelerate gene function analysis could be increasingly feasible for a wide range of microorganisms. In addition, as these databases grow the chance of sampling closely related isolates that differ at the locus of interest will grow with them. To the extent isolates (or phenotypic measurements thereof) are available with genomic sequences, it will then be possible to go one step further, evaluating the extent to which genetic differences experimentally proven to cause phenotypic traits in laboratory strains are correlated with these traits in natural populations, where genetic backgrounds will be more variable. Analogous to testing animal model results in human populations by epidemiological studies, such surveys of natural genetic and phenotypic variation will provide a much fuller picture of the significance of laboratory findings.

## Conclusions

A single nucleotide change in pneumococcal RelA/ SpoT homolog involved in the stringent response (GTP pyrophosphokinase, TIGR4 locus tag SP_1645) was identified to reduce the ability of pneumococcus to compete for colonization and decrease the resistance to killing by human neutrophils. The identification process was shown to benefit from a strategy that includes analyzing 616 previously published pneumococcal genome sequences to select closely-related strains carrying genetic variation of interest for phenotypic evaluation. The utility of this strategy was additionaly demonstrated by identifing variation in another RelA/ SpoT homolog (TIGR4 locus tag SP_1097) that causes change in resistance to neutrophil-killing. We conclude that analysis of naturally occurring genome diversity can be used to complement traditional genetics approaches to accelerate the genotype-phenotype analysis.

## Methods

### Ethics statement

Human blood was obtained from healthy adult volunteers according to a protocol approved by the Office of Human Research Administration at Harvard School of Public Health (protocol number CR-10199-04). All adult subjects provided informed consent in written.

All animal work has been conducted in compliance with the Animal Welfare Act and the guidelines of the U.S. Public Health Service Policy on Humane Care and Use of Laboratory Animals, and specifically approved by the Institutional Animal Care and Use Committee (IACUC) of Harvard Medical School (protocol number 2991; Animal Welfare Assurance of Compliance A3431-01 and AAALAC Accreditation #000009, 6/12/13). Mice were euthanized via CO_2_ inhalation followed by bilateral thoracotomy.

### Strains, cells, and animals

Six capsular variants of the TIGR4 strain (TIGR4:1, TIGR4:4, TIGR4:14, TIGR4:19A, TIGR4:19F, and TIGR4:23F) were constructed and reported previously [4]. The SpnYL101 strain is a laboratory variant of the TIGR4:19F strain, which arose during subcultures of the TIGR4:19F strain. To construct the SpnYL102 and the SpnYL103 strains, the SP_1645 locus in the TIGR4:19F and SpnYL101 was replaced with a Janus-type cassette [17] by using the transformation protocol described previously [18,19]. The Janus cassette in the SpnYL102 strain was then replaced by the allele corresponding to SP_1645 sequence in SpnYL101 to create the SpnYL104 strain. Similarly, the Janus cassette in the SpnYL103 strain was then replaced by the SP_1645 sequence amplified from the TIGR4:19F genomic DNA by PCR to create the SpnYL105 strain.

To construct the SpnYL106 strain, the upstream sequence of the SP_1097 locus (TIGR4 reference genome position 1029105–1030387), the kanamycin-resistance marker, the allele sequence corresponding to SP_1097 in isolate 135771 (including 109-bp upstream of the start codon), and the downstream sequence of the SP_1097 locus (TIGR4 reference genome position 1031171–1032329) were amplified by PCR and the PCR products were ligated in that order by using the Gibson Assembly Kit (New England BioLabs, Ipswich, MA). The assembly product was used to transform TIGR4:6B with selection for kanamycin resistance. After transformation, the SP_1097 locus was amplified by PCR from 12 kanamycin-resistant clones and the PCR products were sequenced to identify clones with correct allele sequence corresponding to SP_1097 in isolate 135771. The same strategy was used to construct the SpnYL107 strain.

Nasopharyngeal carriage isolates were colony-purified prior to use. All strains were grown in Todd Hewitt Broth with 0.5% yeast extract (THY) (BD, Franklin Lakes, NJ) at 37°C with 5% CO_2_. Strains and PCR primers used in this study are listed in Additional file 1: Table S3.

Neutrophils were isolated from human blood using Histopaque 10771, 11191 gradient reagents (Sigma-Aldrich, St. Louis, MO) according to the manufacturer’s instructions and used immediately.

Wild-type C57BL/6 mice were obtained from the Jackson ImmunoResearch Laboratories, Bar Harbor, ME. All mice were female, 9 to 10 weeks old at the start of experiments, and were kept in a BSL2 facility.

### Bacterial growth rate

Strains were streaked onto blood agar plates and cultured at 37°C in 5% CO_2_ overnight. Twelve colonies from each strain were subcultured in Todd–Hewitt medium with 0.5% yeast extract (THY; Becton Dickinson and Company, Sparks, MD) until O.D. 620 reached ~ 0.4 and then diluted into THY medium at a starting culture O.D. of ~0.005. Growth was monitored in sterile flat-bottomed 96-well microtitre plates (Nunc, Denmark) containing 200 μl culture each well every 30 minutes using a VERSAmax microplate reader (Molecular Devices, Sunnyvale, CA) over 6 hours. The growth curves were fitted to an exponential growth equation and the growth rate was estimated using Graphpad Prism software (GraphPad Software, Inc., CA).

### Mouse carriage model and competitive index quantification

In the single-strain colonization experiment, C57BL/6 mice (n = 5 for each group) were inoculated intranasally with in 10 μl of PBS containing approximately 1 × 10^7^ CFU of either TIGR4:19F or SpnYL101. After inoculation, nasal wash samples were collected on day 1 by live sampling and on day 4 by post-mortem tracheal wash as previously described [13]. Aliquots of each sample were titered on gentamicin plate (2.5 mg/L) to determine the colony forming unit (CFU) density.

The multi-strain pneumococcal colonization experiments were performed essentially as previously described [20]. In 6-strain colonization experiments, five TIGR4 capsular variants (TIGR4:1, TIGR4:4, TIGR4:14, TIGR4:19A, TIGR4:23F) were mixed with either TIGR4:19F or SpnYL101 at equal ratio. C57BL/6 mice were inoculated intranasally with the mixtures in 10 μl of PBS containing approximately 1 × 10^6^ CFU of each strain. In 2-strain colonization experiments, a reference strain (TIGR4:19A) was mixed with either SpnYL104 or SpnYL105 at equal ratio. C57BL/6 mice were inoculated intranasally with the mixtures in 10 μl of PBS containing approximately 5 × 10^6^ CFU of each strain. Nasal wash samples were collected up to 7 days after challenge as previously described [13]. Aliquots of each sample were titered to determine the colony forming unit (CFU) density in sample. The remaining samples were cultured overnight on blood agar plates supplemented with gentamicin to a final concentration of 2.5 mg/L, and all bacterial growth was harvested for genomic DNA extraction.

Genomic DNA was purified from cultures of samples collected from animals using DNeasy Blood and Tissue kit (QIAGEN, Valencia, CA). The relative abundance of each strain in a sample was determined by a relative quantification protocol of the 7300 Real Time PCR System (Applied Biosystems). Serotype-specific primers were adopted from a previous study [21] and are listed in Additional file 1: Table S3. The calibrator was composed of 6 types of genomic DNA (TIGR4:1, TIGR4:4, TIGR4:14, TIGR4:19A, TIGR4:19F, and TIGR4:23F) with equal concentration (0.25 ng/μl each). The DNA sequence specifically amplified by primers 19 F-forward and 19 F-reverse was used as the endogenous control while DNA sequences amplified by other serotype-specific primers were treated as targets. Total reaction volume of 25 μl was composed of 1 × SYBR GREEN PCR Master Mix (Applied Biosystems), 2 ng of genomic DNA and 400 nM of each primer. Two replicates reactions for each sample and the calibrator were performed. The relative level of each target in a sample was calculated by the RQ Study software (Applied Biosystems) according to manufacturer’s protocol. The frequency of the TIGR4:19F or SpnYL101 specific DNA in a sample was calculated as 1/(1+ the sum of all targets level). The competitive index of a 19 F variant was calculated as A_1_-A_0_, where A_1_ and A_0_ are the log_10_ transformation of the ratio of the 19 F specific DNA frequency to the (1-19 F specific DNA frequency) in a nasal wash sample and in the inoculation mixture, respectively. This relative quantification method has been validated by using DNA samples of known composition (Additional file 1: Figure S3 and Table S4). According to the validation, we set 3 and −3 as the high and low detection limits for the competitive index, respectively. Calculated competitive index values beyond the limit of detection were rounded to the nearest limit value.

Competitive indexes for strains SpnYL106 and SpnYL107 were measured against a reference strain of the same serotype but distinct antibiotic resistance. The reference strain, SpnYL049 (serotype 6B, Kanamycin sensitive (Kan^s^) and Trimethoprim resistant (Tri^r^)), was mixed with either SpnYL106 (serotype 6B Kan^r^Tri^s^) or SpnYL107 (serotype 6B, Kan^r^ Tri^s^) at equal ratio. C57BL/6 mice (n = 10 for each group) were inoculated intranasally with the mixtures in 10 μl of PBS containing approximately 5 × 10^6^ CFU of each strain. After inoculation, nasal wash samples were collected on day 1 by live sampling and on day 4 by post-mortem tracheal wash as previously described [13]. Aliquots of each sample were titered on two types of blood agar plates: Tri plate (containing 3.2 mg/mL Trimethoprim and 2.5 mg/L gentamicin) and Kan plate (containing 500 mg/L Kanamycin). Titer below the detection limit was denoted as one-half on the detection limit. For each sample in which at least one strain was detectable, the competitive index was calculated as: log_10_(Kan^r^ CFU density/Tri^r^ CFU density) in the sample - log_10_(Kan^r^ CFU density/Tri^r^ CFU density) in the inoculation mixture.

### Neutrophil surface killing assay

Neutrophil surface killing assays were performed as described previously [4]. Briefly, bacteria were grown to mid-log phase and frozen in THY/10% glycerol at −80°C. On the day of the experiment, bacteria were thawed and diluted to 5 × 10^3^ CFU/mL in saline, and 10 μL of this suspension was spotted and allowed to dry at room temperature on trypticase soy agar with 5% defibrinated sheep blood, with 10 replicates per plate. Twenty microliters of neutrophils (2 × 10^6^ cells/mL) were then overlaid, allowed to dry, and incubated overnight at 37°C with 5% CO_2_. Percent survival was calculated by comparing killing of each strain to a duplicate control plate with no neutrophils.

### Whole-genome sequencing

Genomic DNA from isolates TIGR4:19F and SpnYL101 were used to generate multiplexed Illumina libraries using Nextera DNA sample preparation kits. These were sequenced on the Illumina MiSeq platform to produce 151 nt paired-end reads, generating a total of 213,083 reads (64.4 Mb of data) for TIGR4:19F and 383,055 reads (115.7 Mb of data) for SpnYL101. This equates to over 30-fold and over 50-fold mean coverage of a typical pneumococcal genome, respectively. Illumina sequence data were mapped against the complete genome of *S. pneumoniae* TIGR4 (GenBank accession: AE005672) as paired end reads using SMALT v0.6.1. Bases were called using Samtools [22] and VCFtools [23] using the criteria described previously [24]. These sequence data have been submitted to the Sequence Read Archive (SRA) with accession number SRX535517.

### *In silico* screen of carriage isolates

The collection of carriage isolates used in this study correspond to 616 *de novo* assemblies of asymptomatically carried *S. pneumoniae* isolates, in which 5,442 clusters of orthologous genes (COGs) were identified and were previously reported [9]. BLASTP was used to identify the COG corresponding to each TIGR4 gene that is affected by SNPs between TIGR4:19F and SpnYL101. To identify an isolate pair varied substantially at the target gene, coding sequences in the COG were aligned by CLUSTAL 2.1 [25]. After alignment, two sequences showed the least percent identity at the target gene and belonged to isolates of the same serotype were chosen to form a pair. In case of a tie (two or more serotype-matched pairs with equally low within-pair sequence identity), the pair that represents the closest relatives, as ascertained through the shortest phylogenetic distance calculated from a whole genome alignment [9], was used in subsequent phenotypic evaluations. A flowchart of the porecess is shown in Additional file 1: Figure S4.

### Polysaccharide quantification

Cell-associated type 19 F capsular polysaccharide was quantified based on a previously published inhibition ELISA protocol [4,26]. Briefly, immunolon ELISA plates (Thermo Scientific, Waltham, MA) were coated by incubating with 5 μg/mL type 19 F capsular polysaccharide (ATCC, Manassas, VA; 100 μL/well) overnight. Either standard dilutions of type 19 F polysaccharide or serial dilutions of mid-log phase bacteria were mixed 1:1 with typing serum 19b (Statens, 1:5000–1:10,000). The mixtures were incubated in the coated ELISA plates for two hours. Typing sera captured by the coated plates were detected with goat anti-rabbit IgG-HRP (Pierce, 1:60,000) and the TMB developing substrate (KPL, Gaithersburg, MD). After developing was terminated by addition of 1 N HCl, the absorbance at 450 nm was measured using a VERSAmax microplate reader (Molecular Devices, Sunnyvale, CA). The software accompanied the microplate reader was used to calculate the capsular polysaccharide concentration in each sample by a standard-curve method (4-parameter fit).

### Selection of putitative pneumococcal SR pathway genes

To select a pneumococcal gene putatively corresponding to Lon in the SR pathway, a text search of “PDZ domain-containing protein” in KEGG ORTHOLOGY (http://www.genome.jp/kegg/) identified orthologous group K07177, from which the only gene found in *S. pneumoniae* TIGR4 strain, SP_1967, was chosen. Similarly, to select a pneumococcal gene putatively corresponding to rpoS in the SR pathway, a text search of “RNA polymerase primary factor” in KEGG ORTHOLOGY identified orthologous group K03086, from which the only gene found in *S. pneumoniae* TIGR4 strain, SP_1073, was chosen. To select a pneumococcal gene putatively corresponding to spoT in the SR pathway, a text search of “putative GTP pyrophosphokinase” in KEGG ORTHOLOGY identified orthologous group K03086, from which the only gene found in *S. pneumoniae* TIGR4 strain, SP_1073, was chosen.

### Statistics

Competitive index between two strains was compared using a Mann–Whitney test. Survival rate, growth rate, or capsular polysaccharide production between two strains was compared using a t-test. All p-values were calculated for two-tailed tests and p < 0.05 was considered as significant. Statistical analyses were conducted using the GraphPad Prism V5.0 software (GraphPad Software, San Diego, CA, USA) and the R software package (http://www.r-project.org/).

## Additional file

Additional file 1: Figure S1. Growth rate of TIGR4:19F and the SpnYL101. The strains were cultured in THY medium at 37°C with initial O.D. 620 around 0.005, and the increase in O.D. 620 were measured for 2 hours. The increase in O.D. was fitted to an exponential growth to estimate the growth rate. Error bars represent 95% CI. The p-value is derived from t-test (n = 12 for each group). **Figure S2.** Competitive indexes of strains SpnYL106 and SpnYL107 during mouse colonization were measured against a reference strain SpnYL049, as described in Materials and Methods. Competitive indexes derived from mousse nasal wash samples in which at least one strain was detectable are shown. Horizontal bars are medians. p-values are derived from Mann–Whitney tests. **Figure S3.** Validation of the relative quantification assay. A sample was prepared by mixing 6 types of genomic DNA at varied amount to a final total DNA concentration of 2 ng/μl. The input frequency represents the relative abundance of each type of DNA in a sample. The sample was analyzed by the relative quantification assay as described in the Materials and Methods. A linear relationship between the input frequency and the measured frequency is shown. Each dot represents the result of a serotype form the indicated input frequency. The dashed line represents the regression line. Data from 4 independent samples are shown (see Table S4 for detailed data). Slope and goodness of fit (R2) of the regression line are shown. **Figure S4.** Flowchart of the process to identify an isolate pair from a collection of isolates. **Table S1.** Pair wise strain distance derived from previously published data. **Table S2.** Distribution of identified SNPs in a collection of carriage isolates. **Table S3.** Bacterial strains and primers used in this study. **Table S4.** Validation of the relative quantification assay.
