# Supplementary material for: Identification of pneumococcal colonization determinants in the stringent response pathway facilitated by genomic diversity
Source: BMC Genomics. 2015 May 9;16(1):369. doi: 10.1186/s12864-015-1573-6 (PMC4424882; doi:10.1186/s12864-015-1573-6)
Supplement: Additional file 1: Figure S1. — Growth rate of TIGR4:19F and the SpnYL101. The strains were cultured in THY medium at 37°C with initial O.D. 620 around 0.005, and the increase in O.D. 620 were measured for 2 hours. The increase in O.D. was fitted to an exponential growth to estimate the growth rate. Error bars represent 95% CI. The p-value is derived from t-test (n = 12 for each group). Figure S2. Competitive indexes of strains SpnYL106 and SpnYL107 during mouse colonization were measured against a reference strain SpnYL049, as described in Materials and Methods. Competitive indexes derived from mousse nasal wash samples in which at least one strain was detectable are shown. Horizontal bars are medians. p-values are derived from Mann–Whitney tests. Figure S3. Validation of the relative quantification assay. A sample was prepared by mixing 6 types of genomic DNA at varied amount to a final total DNA concentration of 2 ng/μl. The input frequency represents the relative abundance of each type of DNA in a sample. The sample was analyzed by the relative quantification assay as described in the Materials and Methods. A linear relationship between the input frequency and the measured frequency is shown. Each dot represents the result of a serotype form the indicated input frequency. The dashed line represents the regression line. Data from 4 independent samples are shown (see Table S4 for detailed data). Slope and goodness of fit (R2) of the regression line are shown. Figure S4. Flowchart of the process to identify an isolate pair from a collection of isolates. Table S1. Pair wise strain distance derived from previously published data. Table S2. Distribution of identified SNPs in a collection of carriage isolates. Table S3. Bacterial strains and primers used in this study. Table S4. Validation of the relative quantification assay. [file 12864_2015_1573_MOESM1_ESM.pdf]

Figure S1

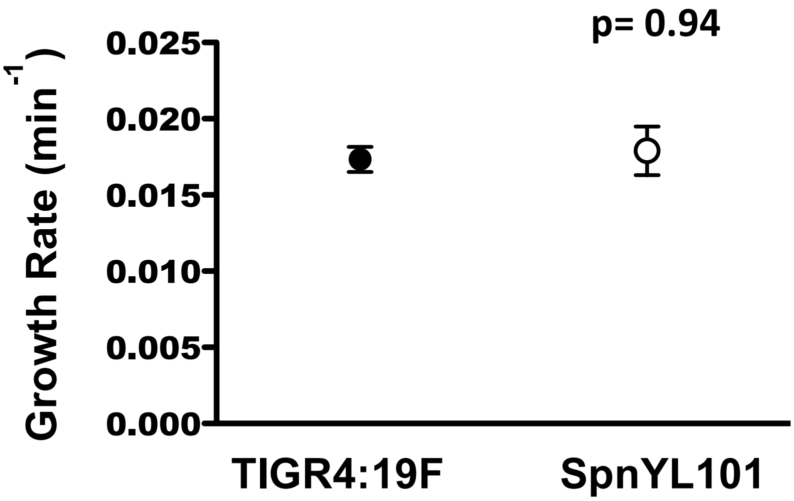

Figure S1. Growth rate of TIGR4:19F and the SpnYL101. The strains were cultured in THY medium at 37 °C with initial O.D. 620 around 0.005, and the increase in O.D. 620 were measured for 2 hours. The increase in O.D. was fitted to an exponential growth to estimate the growth rate. Error bars represent 95% CI. The p-value is derived from t-test (n=12 for each group).

Figure S2

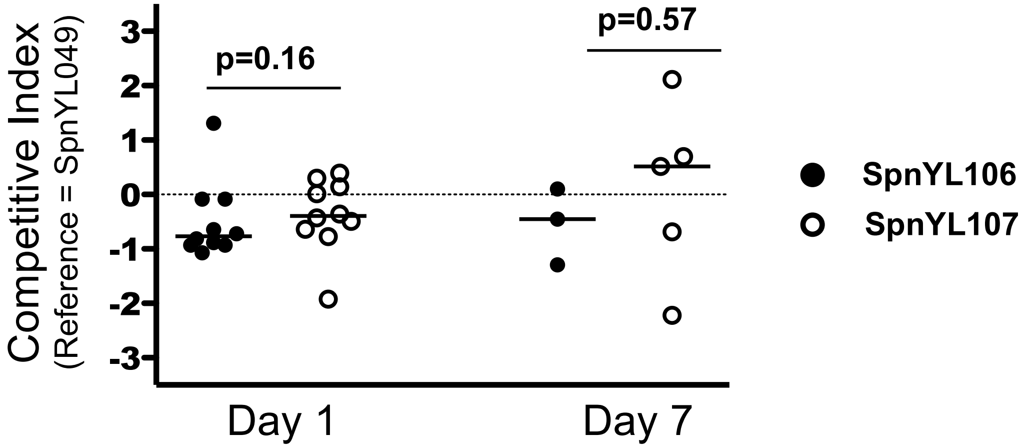

Figure S2. Competitive indexes of strains SpnYL106 and SpnYL107 during mouse colonization were measured against a reference strain SpnYL049, as described in Materials and Methods. Competitive indexes derived from mousse nasal wash samples in which at least one strain was detectable are shown. Horizontal bars are medians. p-values are derived from Mann-Whitney tests.

Figure S3

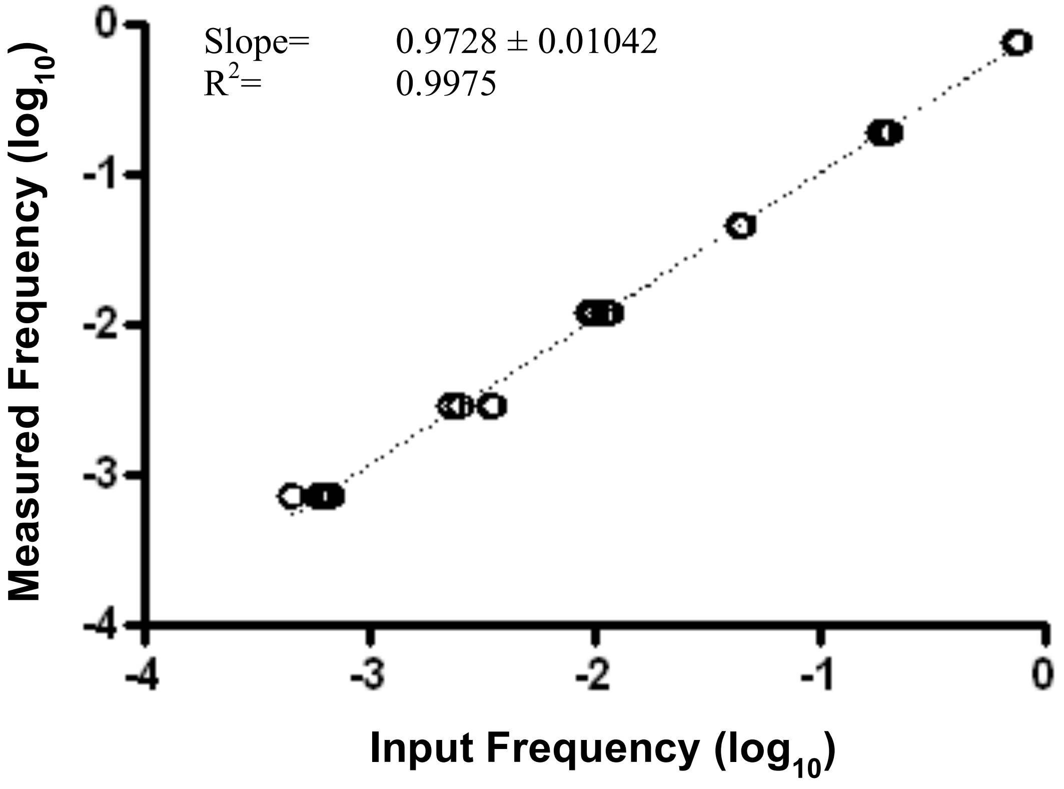

Figure S3. Validation of the relative quantification assay. A sample was prepared by mixing 6 types of genomic DNA at varied amount to a final total DNA concentration of 2ng/μl. The input frequency represents the relative abundance of each type of DNA in a sample. The sample was analyzed by the relative quantification assay as described in the Materials and Methods. A linear relationship between the input frequency and the measured frequency is shown. Each dot represents the result of a serotype form the indicated input frequency. The dashed line represents the regression line. Data from 4 independent samples are shown (see Table S4 for detailed data). Slope and goodness of fit (R^2) of the regression line are shown.

Figure S4

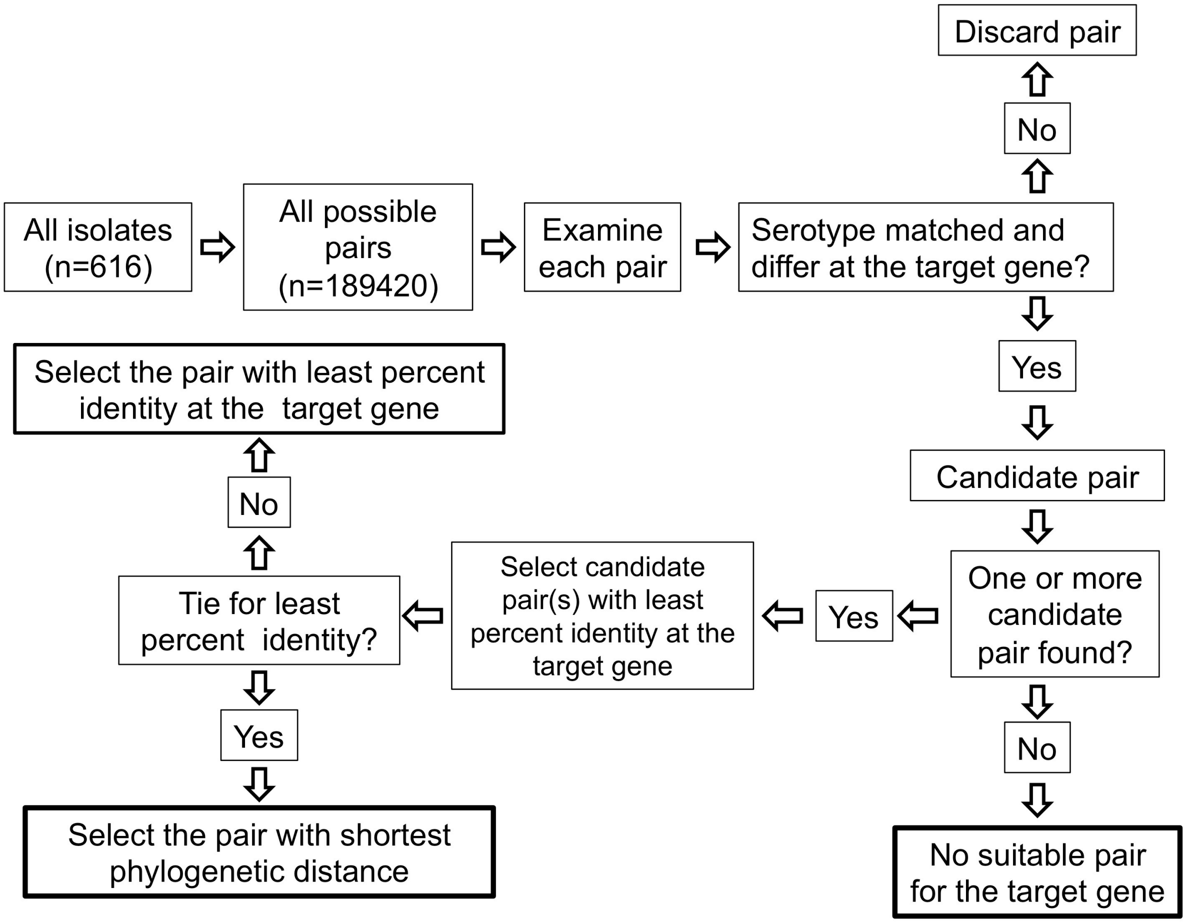

Figure S4. Flowchart of the process to identify an isolate pair from a collection of isolates.

Table S1 Pair wise strain distance derived from previously published data [1]

Isolate1	Isolate 2	Serotype	Separation in core genome phylogeny[a]	Number of distinguishing polymorphic sites[b]
291880	ND6135	6C	5.3×10^{-4}	93
342672	LE4007	6A	8.9×10^{-2}	8718
R34-3067	GL3049	23A	2.1×10^{-4}	28
LE4038	LE4047	19A	5.7×10^{-5}	NA[c]
146066	372297	15B/C	1.4×10^{-4}	24
135771	ND6022	19A	2.3×10^{-4}	22

a. These distances represent the separation between the isolates in the core genome phylogeny reported in [1]
b. Number of sites at which different bases were called in the whole genome alignments reported in [1]
c. No whole genome alignment was generated for this pair of isolates in the original analysis as no appropriate reference genome was available [1]

Table S2 Distribution of identified SNPs in a collection of carriage isolates

TIGR4 Locus tag	Length [a]	SNP sites (%)[b]	SNP in the TIGR4 Gene	Distribution in orthologues (n=616)	
**SP_0152**	693	172 (24.8)	C380A	C=616	A=0
**SP_0655**	2142	501 (23.4)	C906T	C=616	T=0
**SP_1119**	1425	224 (15.7)	G652A	G=615[c]	A=0
**SP_1645**	2223	347 (15.6)	C1019T	C=616	T=0

a Length of DNA alignment of the TIGR4 allele and alleles from the carriage isolates (gaps included)

b Gaps are counted as SNPs

c One of the 616 isolates does not contain a SP_1119 orthologous gene

Table S3. Bacterial strains and primers used in this study

Strain or Primer		Source or reference
S. pneumoniae strains		
TIGR4:1	TIGR4 genetic background with the *cps* locus of a type 1 capsule	[2]
TIGR4:4	TIGR4 genetic background with the *cps* locus of a type 4 capsule	[2]
TIGR4:14	TIGR4 genetic background with the *cps* locus of a type 14 capsule	[2]
TIGR4:19A	TIGR4 genetic background with the *cps* locus of a type 19A capsule	[2]
TIGR4:19F	TIGR4 genetic background with the *cps* locus of a type 19F capsule	[2]
TIGR4:23F	TIGR4 genetic background with the *cps* locus of a type 23F capsule	[2]
SpnYL101	A laboratory variant of the TIGR4:19F	This study
SpnYL102	TIGR4:19F derivative in which the SP_1645 locus is replaced by a Janus cassette	This study
SpnYL103	SpnYL101 derivative in which the SP_1645 locus is replaced by a Janus cassette	This study
SpnYL104	TIGR4:19F derivative in which the SP_1645 locus is replaced by a the SP_1645 locus sequence from the SpnYL101	This study
SpnYL105	SpnYL101 derivative in which the SP_1645 locus is replaced by a the SP_1645 locus sequence from the TIGR4:19F	This study
TIGR4:6B	TIGR4 genetic background with the *cps* locus of a type 6B capsule	[2]
SpnYL049	Trimethoprim resistant TIGR4:6B by selection of mutations	This study
SpnYL106	TIGR4:6B derivative in which the SP_1097 locus is replaced by a kanamycin-resistant marker followed by the allele corresponding to the SP_1097 allele from carriage isolate 135771	This Study
SpnYL107	TIGR4:6B derivative in which the SP_1097 locus is replaced by a kanamycin-resistant marker followed by the allele corresponding to the SP_1097 allele from carriage isolate ND6022	This Study
291880	Asymptomatically carried isolate from MA, USA	[1]
ND6135	Asymptomatically carried isolate from MA, USA	[1]
342672	Asymptomatically carried isolate from MA, USA	[1]
LE4007	Asymptomatically carried isolate from MA, USA	[1]
R34-3067	Asymptomatically carried isolate from MA, USA	[1]
GL3049	Asymptomatically carried isolate from MA, USA	[1]
LE4038	Asymptomatically carried isolate from MA, USA	[1]
LE4047	Asymptomatically carried isolate from MA, USA	[1]
146066	Asymptomatically carried isolate from MA, USA	[1]
372297	Asymptomatically carried isolate from MA, USA	[1]
135771	Asymptomatically carried isolate from MA, USA	[1]
ND6022	Asymptomatically carried isolate from MA, USA	[1]
Primers		
1-forward	CGTGCGGTAATTGAAGCTATGA	[3]

1-Reverse	TGTGGCCCCAGCAACTCT	[3]
4-forward	TGGGATGACATTTCTACGCACTA	[3]
4-Reverse	CCGTCGCTGATGCTTTATCA	[3]
14-forward	CGACTGAAATGTCACTAGGAGAAGAT	[3]
14-Reverse	AATACAGTCCATCAATTACTGCAATACTC	[3]
19A-forward	TTCGACGACGTATCAGCTTCA	[3]
19A-Reverse	TCATTGAGAGCCTTAACCTCTTCA	[3]
19F-forward	GGTCATGCGAGATACGACAGAA	[3]
19F-Reverse	TCCTCATCAGTCCCAACCAATT	[3]
23F-forward	TGCTATTTGCGATCCTGTTCAT	[3]
23F-Reverse	AGAGCCTCCGTTGTTTCGTAAA	[3]

Table S4 Validation of the relative quantification assay[a]

Serotype	Experiment	Input Frequency	Measured Frequency
**1**	1	0.7500	0.7413
**4**	1	0.1880	0.2015
**14**	1	0.0470	0.0434
**19A**	1	0.0120	0.0111
**19F**	1	0.0029	0.0023
**23F**	1	0.0007	0.0005
**1**	2	0.0007	0.0006
**4**	2	0.0029	0.0034
**14**	2	0.0120	0.0094
**19A**	2	0.0470	0.0452
**19F**	2	0.1880	0.1821
**23F**	2	0.7500	0.7593
**1**	3	0.0120	0.0116
**4**	3	0.0029	0.0036
**14**	3	0.0007	0.0007
**19A**	3	0.7500	0.7503
**19F**	3	0.1880	0.1883
**23F**	3	0.0470	0.0456
**1**	4	0.0470	0.0454
**4**	4	0.1880	0.1871
**14**	4	0.7500	0.7545
**19A**	4	0.0007	0.0006
**19F**	4	0.0029	0.0025
**23F**	4	0.0120	0.0099

a Data in this table were used to produce Figure S3. In each experiment, a sample was prepared by mixing 6 types of genomic DNA at varied amount to a final total DNA concentration of 2ng/µl. The input frequency represents the relative abundance of each type of DNA in the sample calculated according to the DNA concentration of each type. The Measured Frequency represents the relative abundance of each type of DNA in the sample derived from the quantification assay in the Materials and Methods. Data from 4 independent experiments are shown.

REFERENCES

1. Croucher NJ, Finkelstein JA, Pelton SI, Mitchell PK, Lee GM, et al. (2013) Population genomics of post-vaccine changes in pneumococcal epidemiology. Nature genetics 45: 656-663.
2. Weinberger DM, Trzcinski K, Lu YJ, Bogaert D, Brandes A, et al. (2009) Pneumococcal capsular polysaccharide structure predicts serotype prevalence. PLoS pathogens 5: e1000476.
3. Azzari C, Moriondo M, Indolfi G, Cortimiglia M, Canessa C, et al. (2010) Realtime PCR is more sensitive than multiplex PCR for diagnosis and serotyping in children with culture negative pneumococcal invasive disease. PloS one 5: e9282.
